# Effect of Two Soybean Varieties Treated with Different Heat Intensities on Ileal and Caecal Microbiota in Broiler Chickens

**DOI:** 10.3390/ani12091109

**Published:** 2022-04-26

**Authors:** Florian Hemetsberger, Benjamin Zwirzitz, Nadia Yacoubi, Wolfgang Kneifel, Karl Schedle, Konrad J. Domig

**Affiliations:** 1 Department of Food Science and Technology, Institute of Food Science, University of Natural Resources and Life Sciences Vienna, Muthgasse 18, 1190 Vienna, Austria; florian.hemetsberger@boku.ac.at (F.H.); benjamin.zwirzitz@boku.ac.at (B.Z.); wolfgang.kneifel@boku.ac.at (W.K.); 2 Department of Agrobiotechnology, Institute of Animal Nutrition, Livestock Products and Nutrition Physiology, University of Natural Resources and Life Sciences Vienna, Muthgasse 11, 1190 Vienna, Austria; karl.schedle@boku.ac.at; 3 Evonik Operations GmbH, 45128 Essen, Germany; nadia.yacoubi@evonik.com

**Keywords:** soybean variety, heat treatment, broiler, microbiota, ileum, caecum, 16S rRNA amplicon sequencing

## Abstract

**Simple Summary:**

Soybeans are an essential part of today’s poultry nutrition diets because of their high protein content and quality. To ensure optimum digestibility in monogastric animals, soybeans need to be thermally processed. As required heat intensities depend on individual soybean properties, the emergence of highly heterogenic soybean batches is a challenge for adequate processing conditions. Molecular changes occurring during heat treatment can alter the microbial communities colonizing the animals’ guts. Gut microbiota is of great importance for both its host animal’s performance and health. To investigate the effect of heat treatment and soybean variety on the chickens’ microbiota, two soybean varieties were selected, treated at two different heat intensities and subjected to a feeding trial. DNA was then extracted and sequenced to identify different bacterial populations in the digesta of certain gut sections. Results showed that both the soybean variety and the applied heat treatment affected the abundance of certain bacterial species in the gut of chickens, but no effect on the taxonomy level of family or genus appeared. This underlines the sensitivity and reactivity of the highly complex microbial community to apparently small dietary differences.

**Abstract:**

Soybean products are of high importance for the protein supply of poultry. Heat treatment of soybeans is essential to ensure optimal digestibility because of intrinsic antinutritive factors typical for this feed category. However, excessive treatment promotes the Maillard reaction and reduces protein digestibility. Furthermore, Europe’s efforts are to decrease dependence on imports of soybean products and enlarge local production. This process will include an increase in the variability of soybean batches, posing great challenges to adequate processing conditions. Intrinsic soybean properties plus heat treatment intensity might be able to modulate the gut microbiota, which is of crucial importance for an animal’s health and performance. To assess the influence of heat treatment and soybean variety on gut microbiota, 2 soybean cakes from 2 varieties were processed at 110 °C or 120 °C and subsequently fed to 336 one-day-old broiler chickens. After 36 days, the animals were slaughtered, and the digesta of the ileum and caecum was collected. Next, 16S rRNA amplicon sequencing of the extracted DNA revealed a high discrepancy between gut sections, but there were no differences between male and female birds. Significant differences attributed to the different soybean varieties and heat intensity were detected for certain bacterial taxa. However, no effect on specific families or genera appeared. In conclusion, the results indicated the potential of processing conditions and soybean variety as microbiota-modulating factors.

## 1. Introduction

Soybeans are a protein source of high and consistent quality and constant availability and thus comprise a major component in today’s livestock diets. To ensure high nutritional suitability, soybeans or products require adequate processing before feeding monogastric animals. Raw products interfere with the digestive function of the animals because of anti-nutritional products such as trypsin inhibitors [1]. Hence, anti-nutritional properties are to be minimized by tailored heat treatment or other feed processing techniques [2]. However, if applied excessively, heat can also lead to reduced feed quality primarily because of decreasing protein digestibility [3]. In addition to its function as a source of nutrients, the feed can also affect the microbial gut community of animals. Broad insight into microbial diversity driven by today’s culture-independent methods facilitates the chance to correlate animal growth performance or disease susceptibility with particular microbial communities [4]. Gut microbiota is crucial for the development and maintenance of intestinal physiology, immune defense of the host, optimal utilization of feed ingredients, production of vitamins and detoxification of potentially harmful substances [5,6]. It is well-known that microbiota’s quantitative and qualitative composition may vary greatly due to influences such as diet and its ingredients and the environment, host genetics, and health challenges [7]. In this context, diets also possess the ability to heavily impact the microbial gut community by factors such as prebiotics, probiotics and antibiotics, protein concentration, and the fiber content or the physicochemical properties of the diet [8,9]. Additionally, substances generated during the thermal processing of feed affect the gut microbiota, which can even be specific for certain bacterial species [10,11]. A multitude of products of the Maillard reaction type can be generated during the heat treatment of proteinaceous feed ingredients, some of which have been identified as microbiome modulators, although the particular molecules responsible for the effect on bacteria often remain unclear [11,12]. However, several heat-linked properties are responsible for these microbiome-influencing effects. Some Maillard reaction products, such as fructolysine, can be metabolized by gut microbiota and even serve as a source of butyrate [13,14]. Similar reactions might also have adverse effects on the gut microbiota of chickens upon the heat treatment of soybeans, especially when soybeans are over-processed. Additionally, the binding properties of the Maillard reaction products to iron and other elements are considered to be a relevant factor responsible for microbiome-shifting effects [15,16]. Moreover, heat-damaged protein, eluding its digestion in the small intestine, is subsequently fermented in more distal parts by adjacent organisms [9]. Last but not least, soybeans or other feed commodities of different varieties, regions and growing conditions usually exhibit different responses to heat treatments [17,18]. This is especially important considering that European politics aim to reduce dependency on soybean imports by establishing local production, which might increase the variability of soybean commodities in the future (2017/2116(INI)).

Considering the knowledge gap regarding the interaction of heat-treated soybean-based feed with gut microbiota in poultry, the present study was undertaken to evaluate the effect of two different soybean varieties treated at two different heat intensities on the ileal and caecal microbiota of broilers analyzed by 16S rRNA amplicon sequencing.

## 2. Materials and Methods

### 2.1. Diet and Feeding Trial

Detailed information on the feeding trials and feed components used was published by Hemetsberger et al in Table 1 (2021). Briefly, seven soybean variety samples were characterized in preliminary experiments upon their response to heat treatment. Based on the protein solubility in potassium hydroxide before and after an autoclaving step, two varieties from each end of the spectrum were classified as heat-stable (HS) or heat-labile (HL) varieties. These two varieties showed the highest discrepancy with a loss of 4% and 19% of their initial protein solubility, respectively, and were selected for the feeding trial. Partly de-oiled soybean cakes of the selected varieties were subjected to two different heat intensities through autoclaving, resulting in a 2 × 2 factorial design. Thermal treatments were intended to simulate an optimal (20 min at 110 °C plus 20 min for heating up and cooling down) and an overheated (120 °C for 20 min plus 45 for heating up and cooling down) variant. These experimental soybean cakes were integrated into a broiler diet at a rate of 30%. Diets were calculated to meet the breeder’s requirements (Aviagen, Huntsville, AL, USA, 2017). The 36-day feeding trial was performed with 336 one-day-old chickens (Ross 308) distributed equally among 24 pens, resulting in 6 replicates for each diet. Four representative animals per box (two male and two female animals) were used to collect digesta of the ileum and caecum after slaughtering on the 36th day of the fattening period. Ileal digesta were collected from the proximal 2/3 of the ileum. For digesta samples of the caecum, the contents of both caeca were collected. Samples were collected in 60 mL sterile sampling bags (VWR, Darmstadt, Germany). Digesta samples were stored in solid carbon dioxide immediately after collection and transferred to a −80 °C refrigerator after two days. The experiment was conducted in compliance with the first regulation of keeping animals (BGBI. II Nr. 485/2004). The experiment was reviewed and approved by the Ethics Committee of the University of Natural Resources and Life Sciences, Vienna (reference number 2021/004).

### 2.2. DNA Extraction and Library Preparation

Extracted DNA from the animals’ digesta was used to analyse the gut microbiota via 16S rRNA amplicon sequencing. DNA extraction was performed using the QIAamp PowerFecal Pro DNA Kit (QIAGEN, Hilden, Germany). A total of 0.25 g of thawed digesta was weighed into a dry bead tube with garnet beads from the DNA Isolation Kit. To disintegrate the cell walls, 800 µL of bead solution was added. The samples were heated at 65 °C for ten minutes and vortexed for ten minutes. After centrifugation at 15,000× *g* for one minute, the supernatant was transferred to a 2 mL collection tube. Two hundred microliters of the respective solution of the isolation kit were added, and it was vortexed for five seconds to precipitate non-DNA organic and inorganic material. The tubes were centrifuged at 15,000× *g* for one minute, and the supernatant was transferred to a clean 2 mL microcentrifuge tube. Then, 600 µL of a high-concentrate salt solution was added following 5 s of vortexing; 650 µL of the lysate was loaded onto an MB spin column and centrifuged for one minute. Flow-through was discarded, and the column was centrifuged again. The spin column was transferred to a clean collection tube, and 500 µL of a wash solution was added, followed by a centrifugation step. This step was repeated with 500 µL of an ethanol-based wash solution. Flow-throughs were discarded after each centrifugation. Centrifugation for two minutes ensured the absence of remaining washing solutions. The column was placed on a new 1.5 mL elution tube. Fifty microliters of elution buffer were placed on the column, and DNA was eluted via centrifugation for one minute. The DNA concentration was quantified fluorometrically using Qubit 2.0 (Life Technologies, Carlsbad, CA, USA). In addition to the samples, the DNA was extracted from mock communities (ZymoBIOMICS Microbial Community DNA Standard (D6306) and ZymoBIOMICS Microbial Community DNA Standard II (D6311), Zymo Research Corp., Irvine, CA, USA) following the same protocol. Mock communities were sequenced and analysed next to samples as internal quality control (see Supplementary Materials). Library preparation was performed for two gut sections of 96 animals, resulting in a total of 192 samples. In the first PCR step, the V3 to V4 region of the bacterial 16S rRNA gene was amplified using HPLC purified primers of Microsynth AG (Balgach, Switzerland): NGS_341F_ill (5′-TCG TCG GCA GCG TCA GAT GTG TAT AAG AGA CAC CTA CGG GJG GCW GCA G-3′) and NGS_802R_ill (5′-GTC TCG TGG GCT CGG AGA TGT GTA TAA GAG ACA GGA CTA CHV GGG TAT CTA ATC C-3′). The polymerase chain reaction was performed using the following preparation: 12.5 µL Q5 High-Fidelity 2X Master Mix (New England BioLabs Inc., Ipswich, MA, USA), 5 µL NGS_341F_ill primer (1 µM), 5 µL NGS_802R_ill primer (1 µM) and 2.5 µL of extracted caecal or ileal DNA. PCR was performed using the following thermocycling program: initial denaturation at 98 °C for 30 s, followed by 30 cycles of denaturation (98 °C for 10 s), annealing (60 °C for 15 s) and extension (72 °C for 20 s). The final extension was conducted at 72 °C for 2 min before the temperature was kept at 4 °C until further processing. The PCR products were purified using the peqGOLD Cycle-Pure Kit (VWR International GmbH, Darmstadt, Germany). The size and quality of the PCR products were checked by electrophoresis in a 2% agarose gel. The DNA concentration was measured using Qubit 2.0 (Life Technologies, Carlsbad, CA, USA). With a second PCR, the barcoded primers Illumina XT Nextera v2 (Illumina, San Diego, CA, USA) with Illumina sequencing adapters were attached to the DNA product of the first PCR. The PCR preparation was as follows: 25 µL Q5 High-Fidelity 2X Master Mix (New England BioLabs Inc. Ipswich, MA, USA), 5 µL Nextera index primer 1 (N7xx, 1 µM), 5 µL Nextera index primer 2 (S5xx, 1 µM), and 1–15 µL amplicon PCR product (resulting in 12 ng DNA in the preparation). Depending on the volume of the amplicon PCR product, 0–14 µL of PCR-grade water was added, to sum up to 50 µL for the total preparation. PCR was performed using the following thermocycling program: initial denaturation at 95 °C for 3 min, followed by 8 cycles of denaturation (95 °C for 30 s), annealing (55 °C for 30 s) and extension (72 °C for 30 s). The final extension was conducted at 72 °C for 5 min before the temperature was kept at 4 °C until further processing. The PCR products were purified and checked via gel electrophoresis, and the DNA concentration was measured as described for the first PCR. The PCR products were adjusted to a concentration of 10 ng/µL, pooled in a single vial and subjected to a final check using gel electrophoresis. For DNA sequencing, samples were sent to Microsynth AG (Balgach, Switzerland).

### 2.3. Bioinformatics and Statistical Analysis

To sequence the V3 and V4 regions of the bacterial 16S rRNA gene, two-step Nextera PCR libraries using the primer pair 341F (5′- CCT ACG GGN GGC WGC AG -3′) and 805R (5′- GAC TAC HVG GGT ATC TAA TCC -3′) were created. Subsequently, one Illumina MiSeq platform and a v3 600 cycle kit were used to sequence the PCR libraries. The produced paired-end reads that passed Illumina’s chastity filter were subjected to demultiplexing using Illumina’s bcl2fastq software version v2.20.0.422. The Illumina adapter residuals and primers were trimmed with cutadapt v3.4 [20]. The trimmed reads were then quality filtered using the filterAndTrim command in dada2 v1.16 [21]. The reads were truncated, where the quality score dropped below 30. The maximum number of Ns was set to 0, and the maximum number of errors was set to 2 for forward and reverse reads. After learning the error rates, the reads were dereplicated. Then, the dada2 inference algorithm was run with pseudopooling, after which the reads were merged. Chimeras were removed using the removeBimeraDenovo command with the consensus method. Finally, the remaining high-quality reads were classified against the SILVA SSU 138.1 database [22].

Microbial community analysis was performed in the R environment [23]. The alpha diversity indices were calculated using a dataset rarefied to the minimum sample size (9912) with the packages vegan v2.5.6 and phyloseq v1.37 [24,25]. Relative abundance and prevalence plots were created with ggplot2 v3.3.2 [26]. Beta diversity was assessed with a principal coordinates analysis based on Bray–Curtis distances, and the relative abundances of individual amplicon sequence variants (ASVs) were illustrated as heatmaps in ampvis2 v2.7.1 [27]. Differential abundance analysis was performed with the package DESeq2 v1.33.4. using standard parameters [28]. Correlations of environmental parameters and the microbiome were calculated using MaAsLin2. Only associations for ASVs with a minimum prevalence of 10% and a minimum relative abundance of 1% were calculated. The variable “Box” was set as a random effect, and the Benjamini–Hochberg procedure was applied as a correction method for computing the q-values.

## 3. Results

### 3.1. Effects on Diet

The different processing procedures led to a change in the color of the resulting soybean cakes (Table 1). Using the CIELAB color space, lower values for L* (lightness) and b* (yellowness) indicate a shift from light to dark and from yellow to blue. Higher processing temperatures led to an increased redness (a*) of the soybean cakes. Increasing temperatures led to lower trypsin inhibitor activities and protein solubility. Diets of the finisher phase showed similar contents of crude protein, amino acids and ammonia. Diets prepared with the heat-stable soybean variety had higher starch contents and lower shares of ether extract, crude sugar and crude fiber.

### 3.2. Effects on Microbiota

Sequencing of the 16S rRNA gene of 192 samples from two gut sections resulted in an average of 38,658 sequences per sample.

**Figure 1:**
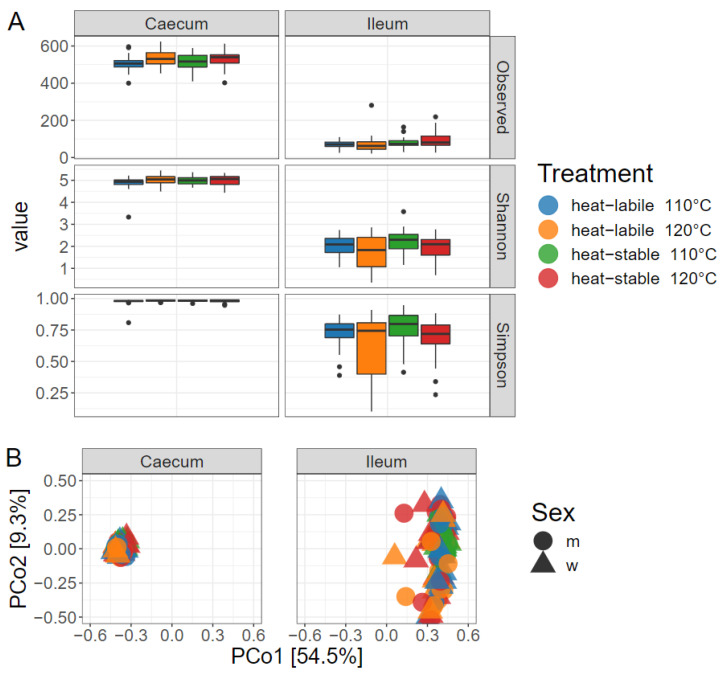
(**A**) Alpha diversity analysis of microbiota in the ileum and caecum of broiler chickens; (**B**) principal component analysis of ileal and caecal microbiota.

Alpha diversity differed significantly between the gut sections (*p* < 0.01). The caecum showed approximately five times more observed reads than the ileum. The Shannon and Simpson diversity indices were higher in the caecum, indicating a higher species richness and evenness (Figure 1A). Feeding the heat-stable soybean variety led to a higher number of observed reads in the ileum (*p* < 0.05). No further differences between heat treatment, variety or sex were observed regarding alpha diversity. Principal component analysis revealed a higher homogeneity between samples in the caecum than in the ileum (Figure 1B). However, beta diversity analysis indicated no differences in heat treatment parameters, variety or sex.

**Figure 2:**
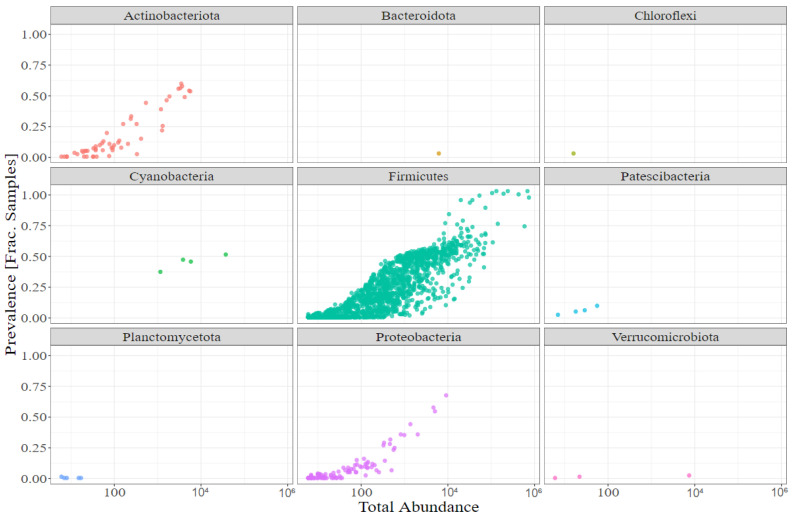
Distribution of phyla across all feeding strategies and gut sections.

A total of nine phyla were observed in the gut of the examined broilers (Figure 2). Firmicutes represented the most abundant phylum, ranging from a minimum of 98.1% (ileum, heat-stable, 110 °C) to 99.5% (ileum, heat-labile, 120 °C) of all observed ASVs. With an average abundance of approximately 0.4% each, Actinobacteria and Proteobacteria were the most frequently occurring phyla after Firmicutes.

**Figure 3:**
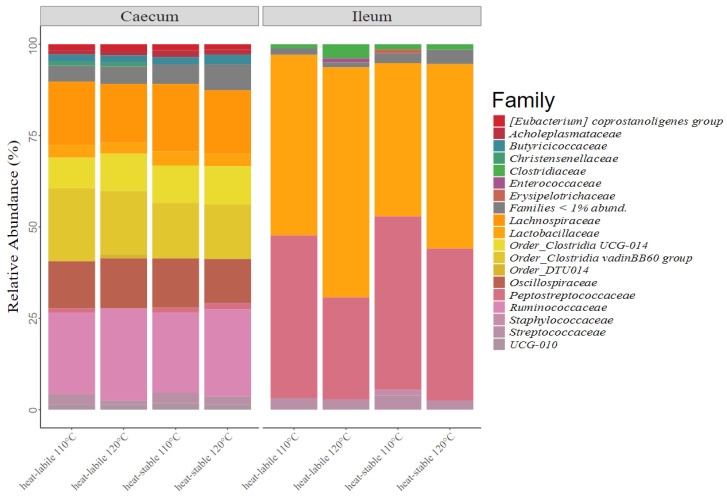
The relative share of taxonomic families with abundances over 1% for all feed regimes in the caecum and ileum.

In total, 18 taxonomic families with relative abundances of over 1% were observed in the caecum and ileum of the studied animals (Figure 3). The Ruminococcaceae, Lachnospiraceae and Clostridia vadinBB60 groups represented the most dominant families in the caecum, with abundances of 23.2%, 17.3% and 16.8%, respectively. Two families dominated the microbiota of the ileum, Lactobaccillaceae (35.4%) and Peptostreprococcaceae (33.4%).

**Figure 4:**
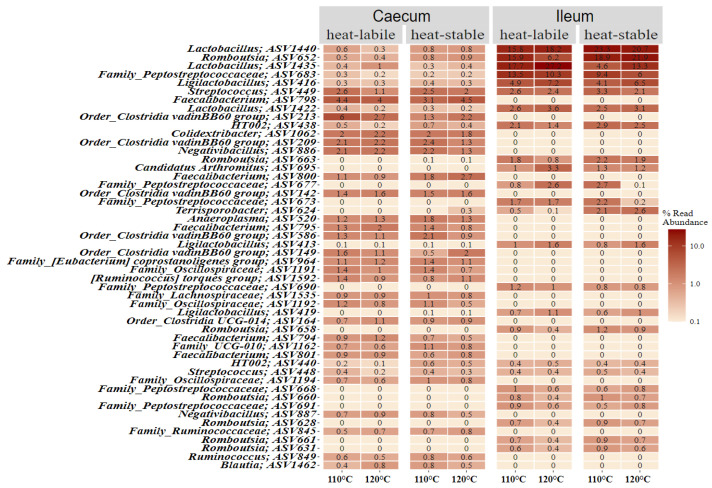
Heatmap displaying the relative abundance of the 50 most frequently occurring amplicon sequence variants. Values are grouped by gut section, soybean variety and heat treatment intensity.

Figure 4 shows the 50 most abundant ASVs in the caecum and ileum with detailed abundances for soybean variety and heat treatment. A small number of ASVs dominated the microbiota of the ileum, mainly members of two orders, Bacilli and Clostridia, which contributed to more than 5% of the relative abundance each. The relative abundance of ASVs in the caecum was more evenly distributed, with none making up more than 6% of reads for any treatment.

**Figure 5:**
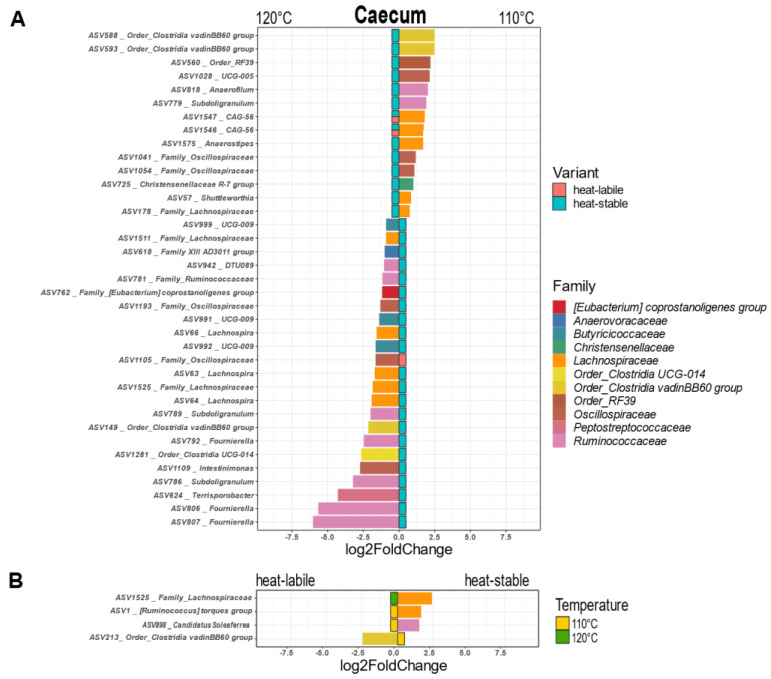
Amplicon sequence variants significantly influenced by heat treatment (**A**) and soybean variety (**B**) in the caecum (*p* < 0.05).

In the caecum, a considerable number of ASVs associated with eleven families were influenced by the intensity of heat treatment of soybeans (Figure 5A). The majority of ASVs were affected only when the heat-stable variety was fed. Soybean processing at 110 °C significantly increased the abundance of members of six families belonging to two phyla (Firmicutes and Tenericutes). Members of nine families (all Firmicutes) were more frequent when 120 °C was applied to soybeans. The abundance of four ASVs was significantly influenced by the soybean variety (Figure 5B). Clostridia vadinBB60 was more abundant with the heat-labile variety, while two members of the family Lachnospiraceae and one member of Ruminococcaceae were more frequent when the heat-stable variety was fed.

**Figure 6:**
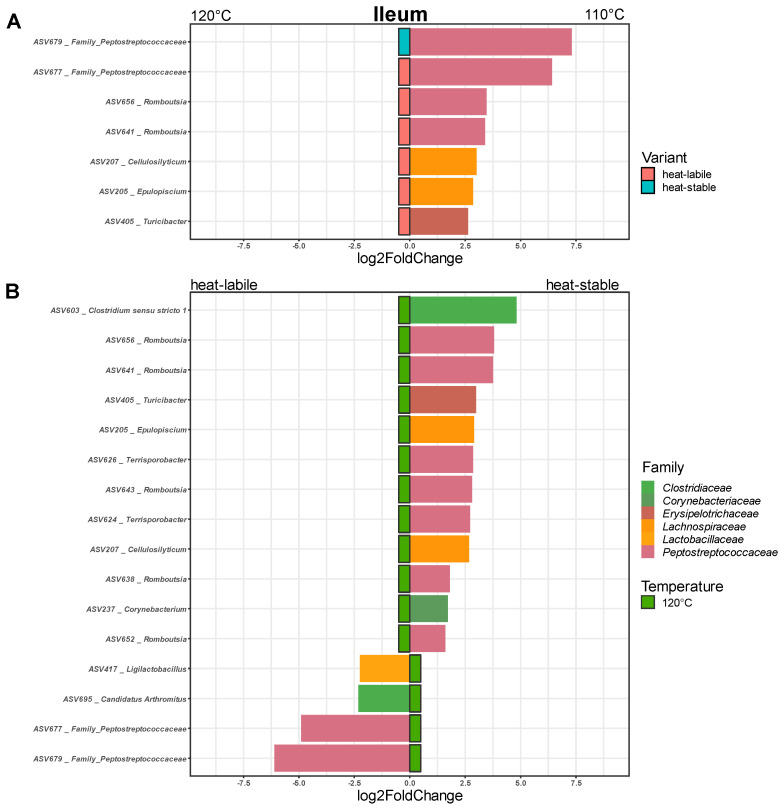
Amplicon sequence variants significantly influenced by heat treatment (**A**) and soybean variety (**B**) in the ileum (*p* < 0.05).

In the ileum, only Firmicutes were affected. The application of 110 °C in soybean treatment led to an increase in the relative abundance of seven ASVs, four members of Peptostreptococcaceae, two Lachnospiraceae and one Erysipelotrichaceae (Figure 6A). The soybean variety affected the abundance of 16 ASVs (Figure 6B). Feeding of the heat-stable variety led to an increased abundance of members of five families. The heat-labile variety caused an increase in two Peptostreptococcaceae, one Clostridiaceae and one Lactobacillaceae.

A correlation analysis of selected parameters received from Hemetsberger et al. (2021) with ASVs found in the ileal and cecal digesta is shown in Figures S1 and S2 in the Supplementary Materials. Used phenotypical parameters were apparent ileal crude protein and amino acid digestibility, nitrogen retention, apparent total tract starch digestibility, weight gain and feed efficiency. Heatmaps show some linkage between certain ASVs and phenotypical data but no significant correlation for taxonomic families or genera.

## 4. Discussion

The observed differences in crude nutrient compositions between the diets arose from the different soybean varieties and exerted some potential influence on gut microbiota. A detailed description of diets on performance indices is given in Hemetsberger et al. (2021). The starch, fat, fiber, and mineral contents of feedstuff are known for their ability to shift the microbial composition in the gastrointestinal tract [29,30,31,32]. Moreover, sugar content is of great relevance as it contributes to the formation of Maillard products. Furthermore, potential antimicrobial substances such as hydroxymethylfurfural can be generated through caramelization during an intensive heat treatment [33]. The effects of certain molecules formed during high-temperature processes on gut microbiota have been described in several studies [34,35,36]. Higher processing temperatures resulted in changes in color, protein solubility and trypsin inhibitor activity; these parameters can be linked to changes in the microbiota. Low protein solubility may result in decreased protein digestibility, which leads to an increased accumulation of crude protein in distal parts of the gastrointestinal tract [37]. On the other hand, high trypsin inhibitor activity can have the same effect due to its impact on protein digestibility [38]. Both aspects can lead to the unfavorable consequence of protein fermentation in the gut [39].

The present study has shown that diversity indices of the microbiota differed to some great extent between the gut sections. As the main fermentation chamber in the avian gastrointestinal tract, the caecum is the segment with the greatest taxonomic diversity and bacterial density resulting from the comparatively long residence time of 12 to 20 h [40,41,42]. In our experiment, the sex of broilers had no significant effect on the microbial composition; however, ambiguous findings can be found in the literature, which indicates a potential minor role of sex on gut microbiota [43,44].

Firmicutes are the most abundant phylum in the intestine of chickens, followed by Bacteroidetes, Proteobacteria, Actinobacteria and Tenericutes [45]. The small intestine is typically dominated by Lactobacillus, Enterococcus and various Clostridiaceae. The caeca of chickens exposed to the slowest passage rate harboring the highest bacterial densities are dominated by Clostridiales. In general, a high Firmicutes to Bacteroidetes ratio is associated with the high energy efficiency of the diet [46]. High microbial diversity and the absence of certain bacteria, such as *Escherichia coli*, can also have a positive correlation with the performance of broilers [4,47]

Across all samples, Firmicutes represented the predominant phylum in the chicken, although earlier studies exhibit higher shares of Bacteroidetes and Proteobacteria [45,48]. One explanation for the high percentage of Firmicutes seen in this study might be the presence of titanium dioxide (TiO_2_), used as an inert digestibility marker in all finisher diets. This assumption agrees with other studies also indicating an increased abundance of Firmicutes in rodents in the presence of TiO_2_ [49]. Although the effects of TiO_2_ nanoparticles on gut microbiota and its metabolism have been discussed extensively, the precise mechanisms of these effects are still not elucidated [50]. However, the induction of oxidative stress via the formation of oxidative reaction species seems to be of crucial importance [51]. Considering all these issues, concealment of a potential dietary effect caused by the high TiO_2_ levels in the applied diets cannot be excluded.

The ileal microbiota is dominated by Lactobacillaceae, which include known beneficial species, and Peptostreptococcaceae [52,53]. The caecum revealed a higher diversity at the family level. The dominating families Oscillospiraceae, Clostridia UCG-014 group, Ruminococcaceae, Lachnospiraceae and Clostridia vadinBB60 group are all members of the class Clostridia, which is known for their important role in carbohydrate fermentation and short-chain fatty acid production [46].

Most changes in the relative abundance of amplicon sequence variants (ASV) were observed in the caecum when the heat-stable variety was fed. However, no explicit shift caused by low or high processing temperatures for any specific taxonomic family could be identified. This promotes the assumption that the effect is rather species or even strain-specific. It remains ambiguous why the effect was mainly restricted to one soybean variety. Irrespective of heat treatment, soybean variety and associated differences between different nutrient contents had little effect, as only four ASVs showed significant changes. In the ileum, processing temperatures of 110 °C increased the frequency of six members of the class Clostridia, regarded as beneficial because of its ability to utilize plant polysaccharides, and one Erysipelotrichia, also a typical colonizer of a chicken’s gut [54,55]. Other than in the caecum, the variety impacted a high number of ASVs, which only appeared for the 120 °C variant. Most of the affected ASVs were represented by the family Peptostreptococcaceae, but no clear allocation to a certain soybean variety could be identified.

## 5. Conclusions

The detailed investigation of gut microbiota has been fueled by the development of modern DNA analysis methods. In the present study, it was shown that the use of different soybean varieties involving different compositional details as well as the application of different heat treatments induces some marked effects on certain members of the gut microbiome. While the different diets had no significant effect on the taxonomic ranks of the genus, family or higher, major differences between ileum and caecum were observed. A separate evaluation of male and female broilers revealed no difference.

## Figures and Tables

**Table 1:** Selected characteristics of the soybean cake and diets of starter, grower and finisher phase. For a detailed description of diets, see Hemetsberger et al. (2021) [19].

Phase	Starter				Grower				Finisher			
Variety	Heat-Stable		Heat-Labile		Heat-Stable		Heat-Labile		Heat-Stable		Heat-Labile	
Heat Treatment	Low	High	Low	High	Low	High	Low	High	Low	High	Low	High
Ingredients, g/kg.												
Corn	486.6		445.6		502.0		461.0		532.3		491.3	
Soybean meal HP	129.0		165.3		111.3		147.5		63.8		100.1	
HS low T	300.0				300.0				300.0			
HS high T		300.0				300.0				300.0		
HL low T			300.0				300.0				300.0	
HL high T				300.0				300.0				300.0
Sunflower oil	17.0		23.2		27.1		33.3		39.8		46.0	
Lignocellulose	10.0		10.0		10.0		10.0		10.0		10.0	
Supplements ^1^	57.4		55.9		49.6		48.2		50.1		48.6	
Titanium dioxide	-		-		-		-		4.0		4.0	
Analyzed composition, g/kg												
Dry matter	891.9	891.6	894.6	896.2	890.9	890.3	895.6	894.9	893.9	894.4	899.0	899.2
Crude protein	239.4	233.4	237.7	236.5	216.5	225.4	220.6	228.0	205.4	205.6	203.1	208.0
Starch	352	357	328	332	352	366	344	339	383	379	356	347
Ether extract	75.4	75.7	78.1	79.8	84.1	82.3	90.4	87.9	98.7	97.9	102.3	102.5
Sugar	41	40	51	46	43	40	47	46	36	36	45	46
Crude fiber	27.8	31.2	34.6	44.0	27.3	28.4	34.0	40.8	29.1	29.5	34.9	32.6
Ash	65.5	65.9	67.2	67.1	62.9	60.0	64.3	63.2	63.3	66.7	68.7	65.5
Calcium	10.6	12.8	12.4	13.0	10.6	10.8	10.8	10.4	12.7	12.3	13.3	12.0
Phosphor	6.4	6.7	6.7	6.8	6.0	6.3	5.4	5.9	5.7	5.9	5.8	5.8
Sodium	1.5	1.4	1.4	1.7	1.5	1.4	1.4	1.2	1.5	1.4	1.6	1.4
Potassium	9.7	9.9	10.0	10.5	8.7	8.9	9.3	9.6	8.3	8.3	9.1	9.4
TIA	1.5	0.8	2.1	0.8	1.5	0.8	2.1	0.9	1.6	0.6	1.8	0.8
Lysine	-	-	-	-	-	-	-	-	13.1	13.1	13.6	16.6
Methionine	-	-	-	-	-	-	-	-	6.2	6.1	6.4	6.4
Threonine	-	-	-	-	-	-	-	-	8.7	8.8	9.0	9.3
Arginine	-	-	-	-	-	-	-	-	13.5	13.5	13.3	13.6
NH_3_	-	-	-	-	-	-	-	-	4.0	4.1	3.9	4.1
Soybean cake characteristics												
L ^2^					82	75	82	74				
a ^2^					2.3	6.2	2.4	6.7				
b ^2^					23.2	20.5	20.8	19.1				
protein solubility ^c^					81%	61%	78%	48%				

Abbreviations: HS, heat-stable variety; HL, heat-labile variety; T, temperature; TIA, trypsin inhibitor activity; ^1^ calcium carbonate, dicalcium phosphate, sodium chloride, synthetic amino acids, minerals/vitamin premix, choline-Cl, phytase, titanium dioxide; ^2^ axes of CIELAB color space L, black-white; a, green-red; b, blue-yellow; ^c^ protein solubility in 2 g/kg potassium hydroxide.

## Data Availability

Raw sequence reads are available on the European Nucleotide Archive under the accession number PRJEB51483.

## Supplementary Materials

The following supporting information can be downloaded at: https://www.mdpi.com/article/10.3390/ani12091109/s1, Figure S1: Heatmap illustrating significant changes of microbial abundance in ileum samples correlating with environmental parameters. The effect size depicts the negative log of the q-value multiplied by the coefficient sign. Taxonomy of the ASVs is indicated at the genus levels or at the lowest rank that could be assigned confidently (Bootstrap support above 50). Only significant changes are shown. Heatmap illustrating significant changes of microbial abundance in ileum samples correlating with environmental parameters. The effect size depicts the negative log of the q-value multiplied by the coefficient sign. Taxonomy of the ASVs is indicated at the genus levels or at the lowest rank that could be assigned confidently (Bootstrap support above 50). Only significant changes are shown.; Figure S2: Heatmap illustrating significant changes of microbial abundance in caecum samples correlating with environmental parameters. The effect size depicts the negative log of the q-value multiplied by the coefficient sign. Taxonomy of the ASVs is indicated at the genus levels or at the lowest rank that could be assigned confidently (Bootstrap support above 50). Only significant changes are shown. Heatmap illustrating significant changes of microbial abundance in caecum samples correlating with environmental parameters. The effect size depicts the negative log of the q-value multiplied by the coefficient sign. Taxonomy of the ASVs is indicated at the genus levels or at the lowest rank that could be assigned confidently (Bootstrap support above 50). Only significant changes are shown.
