# Supplementary figures and images for: Effect of Two Soybean Varieties Treated with Different Heat Intensities on Ileal and Caecal Microbiota in Broiler Chickens
Source: Animals (Basel). 2022 Apr 26;12(9):1109. doi: 10.3390/ani12091109 (PMC9103914; doi:10.3390/ani12091109)

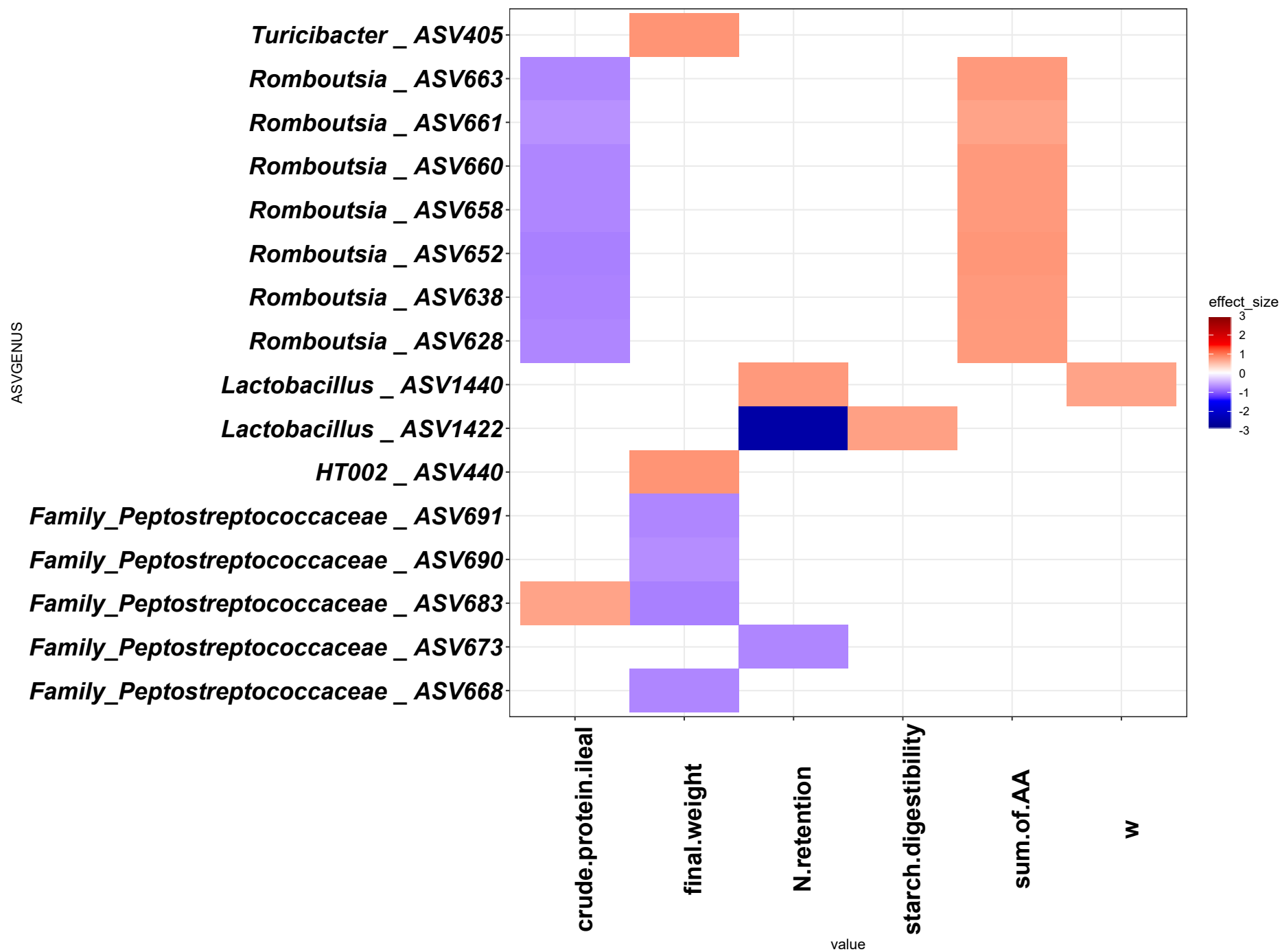

ASVGENUS

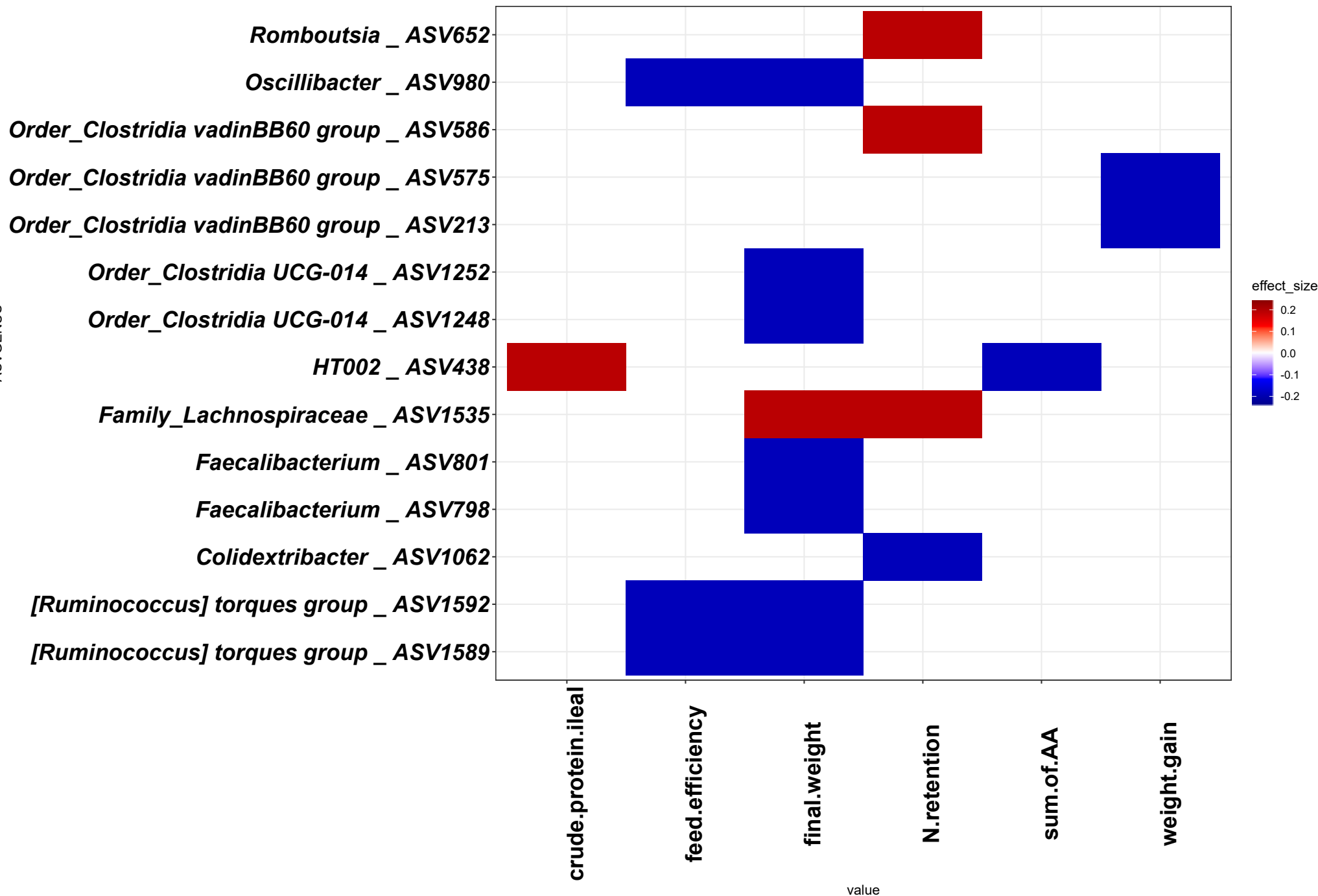

value

Supplement: Supplementary file 1 [file animals-12-01109-s001.zip › animals-1654627-supplementary.pdf]
